# LSM2 drives glioma progression through alternative splicing dysregulation: a multi-omics approach to identify a potential therapeutic target

**DOI:** 10.3389/fonc.2025.1521608

**Published:** 2025-04-29

**Authors:** Cao Yang, Chang Ge, Wenjie Zhang, Jingxuan Xu

**Affiliations:** Department of Neurosurgery, The Second Affiliated Hospital of Xinjiang Medical University, Urumqi, China

**Keywords:** LSM2, glioma, alternative splicing, multi-omics, tumour microenvironment, prognostic biomarker, therapeutic target

## Abstract

**Background:**

Glioma, particularly glioblastoma (GBM), remains a highly aggressive and challenging tumour, characterised by poor prognosis and limited therapeutic options. *LSM2*, an RNA-binding protein, has been implicated in tumour progression, yet its role in glioma remains underexplored. This study aims to investigate the expression, prognostic significance, and molecular mechanisms of *LSM2* in glioma, focusing on its impact on RNA splicing regulation.

**Methods:**

Clinical and transcriptomic data from 163 GBM and 518 lower-grade glioma (LGG) cases from The Cancer Genome Atlas (TCGA) were analysed to assess LSM2 expression and its prognostic value. RNA sequencing was performed on LSM2 knockdown in T98G glioblastoma cells to identify differentially expressed genes (DEGs) and alternative splicing events (ASEs). Bioinformatics tools were employed to perform functional enrichment analyses and construct protein–protein interaction (PPI) networks.

**Results:**

LSM2 expression was significantly elevated in gliomas, particularly in GBM and in tumours with 1p/19q non-deletion or IDH1 mutation (p < 0.001). High LSM2 expression was correlated with shorter overall survival (HR = 1.7, p = 0.01). Knockdown of LSM2 in T98G cells identified 728 upregulated and 1,720 downregulated genes, alongside 1,949 splicing alterations, which primarily affected pathways related to RNA metabolism, DNA damage response, and cell cycle regulation. Key hub genes such as TLN1, FN1, and IRF7 were associated with glioma progression and poor prognosis.

**Conclusion:**

Our findings demonstrate that *LSM2* plays a critical role in glioma progression through the regulation of RNA splicing dynamics. Elevated *LSM2* expression serves as a prognostic biomarker and offers promising potential as a therapeutic target in glioma.

## 1 Introduction

According to the Global Cancer Statistics (2022), worldwide cancer incidence surpassed 20 million new cases with approximately 10 million fatalities, among which GBM – the most aggressive primary malignancy of the central nervous system (CNS) – remains a formidable challenge in neuro-oncology, characterised by an annual incidence of 3–8 cases per 100,000 population and a 5-year survival rate below 5% (1). Despite the marked improvement in prognoses for most solid tumours through multimodal therapeutic regimens (e.g., surgery combined with radiotherapy/chemotherapy) and advancements in targeted therapies and immunotherapeutic approaches, GBM persistently exhibits high recurrence rates and therapeutic resistance (2, 3). This paradox highlights the imperative to elucidate tumour microenvironment heterogeneity. Thus, a deeper understanding of the molecular mechanisms driving glioma progression is essential to identify novel therapeutic strategies.

In recent years, advancements in genetic sequencing, the proliferation of biochips, and the increased accessibility of big data have collectively facilitated the emergence of bioinformatics as a powerful tool for uncovering the mechanisms underlying various diseases (4). By integrating human genomic data, bioinformatics enables the identification of novel biomarkers associated with cancer diagnosis, treatment, and prognosis (5). These developments have significant implications for advancing understanding of glioma pathogenesis, discovering diagnostic biomarkers, and identifying novel therapeutic targets, ultimately prolonging patient survival (6).

In recent years, the role of RNA-binding proteins (RBPs) in regulating post-transcriptional gene expression has gained significant attention. These proteins modulate key cellular processes, including mRNA splicing, stability, and translation, all of which are crucial for maintaining cellular homeostasis (7, 8). Aberrant regulation of RNA metabolism, particularly RNA splicing, is emerging as a key factor in the pathogenesis of various cancers, including glioma. The LSM (Like Sm) protein family, which includes LSM2, plays a pivotal role in RNA splicing, small nuclear RNA (snRNA) processing, and mRNA decay (9). Specifically, LSM2 is part of a hetero-oligomeric complex that facilitates the binding of RNA to small nuclear ribonucleoproteins (snRNPs), ensuring efficient splicing and RNA stability Dysfunction in LSM2 has been implicated in several cancers, such as breast cancer and hepatocellular carcinoma, suggesting its potential as a biomarker and therapeutic target (10, 11). However, there are still few studies on the role of LSM2 in gliomas.

Given the central role of LSM2 in RNA processing and the critical nature of RNA splicing in tumourigenesis, we hypothesise that LSM2 plays a crucial role in glioma progression by modulating RNA splicing events, thereby contributing to tumour aggressiveness and poor patient prognosis. This study aims to investigate the expression of LSM2 in glioma, its prognostic significance, and the molecular mechanisms through which it influences glioma biology, focusing particularly on its role in alternative splicing and its potential as a therapeutic target.

Our research leverages multi-omics approaches, including transcriptomic analysis and RNA sequencing, to systematically examine the expression of LSM2 in gliomas and its association with clinical outcomes. We also explore the functional consequences of LSM2 knockdown in glioblastoma cells, aiming to uncover key genes and pathways regulated by LSM2. By integrating bioinformatics tools for gene enrichment analysis and protein-protein interaction network construction, we aim to identify potential therapeutic targets and provide insights into the molecular mechanisms underlying glioma progression.

## 2 Materials and methods

### 2.1 TCGA data acquisition and analysis

The Cancer Genome Atlas (TCGA, https://portal.gdc.cancer.gov/) is a comprehensive database of tumour genomic data that can be used to identify aberrant values, analyse gene expression discrepancies, and predict co-expressed genes (12). TCGA includes extensive clinical data, such as tumour staging, histopathological classification, grading, and overall survival. We obtained publicly accessible mRNA data and corresponding clinical information from the GBMLGG cohort in TCGA, which comprises 163 cases of GBM and 518 cases of LGG. This dataset was used to analyse the expression pattern and prognostic value of *LSM2* in glioma.

### 2.2 GEPIA analysis for LSM2 expression

Gene Expression Profiling Interactive Analysis (GEPIA; http://gepia.cancer-pku.cn/) was used to perform a pan-cancer analysis of *LSM2* expression across 9,736 tumour samples and 8,587 normal samples sourced from TCGA and GTEx (13). We employed GEPIA for survival analysis and comparison of *LSM2* expression between glioma subtypes.

### 2.3 Human Protein Atlas

HPA (https://www.proteinatlas.org/) is a comprehensive resource platform focusing on the expression, localisation and functional studies of human proteins (14). We used HPA to explore *LSM2* protein expression levels in glioma and normal brain tissues as well as expression in various immune cells.

### 2.4 Tumour Immune Estimation Resource

TIMER (https://cistrome.shinyapps.io/timer/) is an online tool for analysing tumour immune cell infiltration, providing estimates of immune cell infiltration and correlation analyses for multiple cancer types (15). We used it to explore the correlation between LSM2 expression and immune cell infiltration in gliomas.

### 2.5 cBioPortal analysis of LSM2 alteration

cBioPortal for Cancer Genomics (https://www.cbioportal.org/) integrates a multitude of data types, including somatic mutations, DNA copy number alterations, mRNA and miRNA expression, DNA methylation, and protein abundance, providing multi-dimensional visualisation (16). Our analysis incorporated data from TCGA (Firehose Legacy dataset), focusing on two cohorts: LGG (530 cases) and GBM (619 cases). The OncoPrint and Cancer Type Summary modules were employed to investigate the types and frequencies of *LSM2* gene mutations in glioma. Additionally, the Survival module was utilised to assess the prognostic value of genetic alterations.

### 2.6 Cell culture and siRNA-mediated knockdown of LSM2

**Figure 1:**
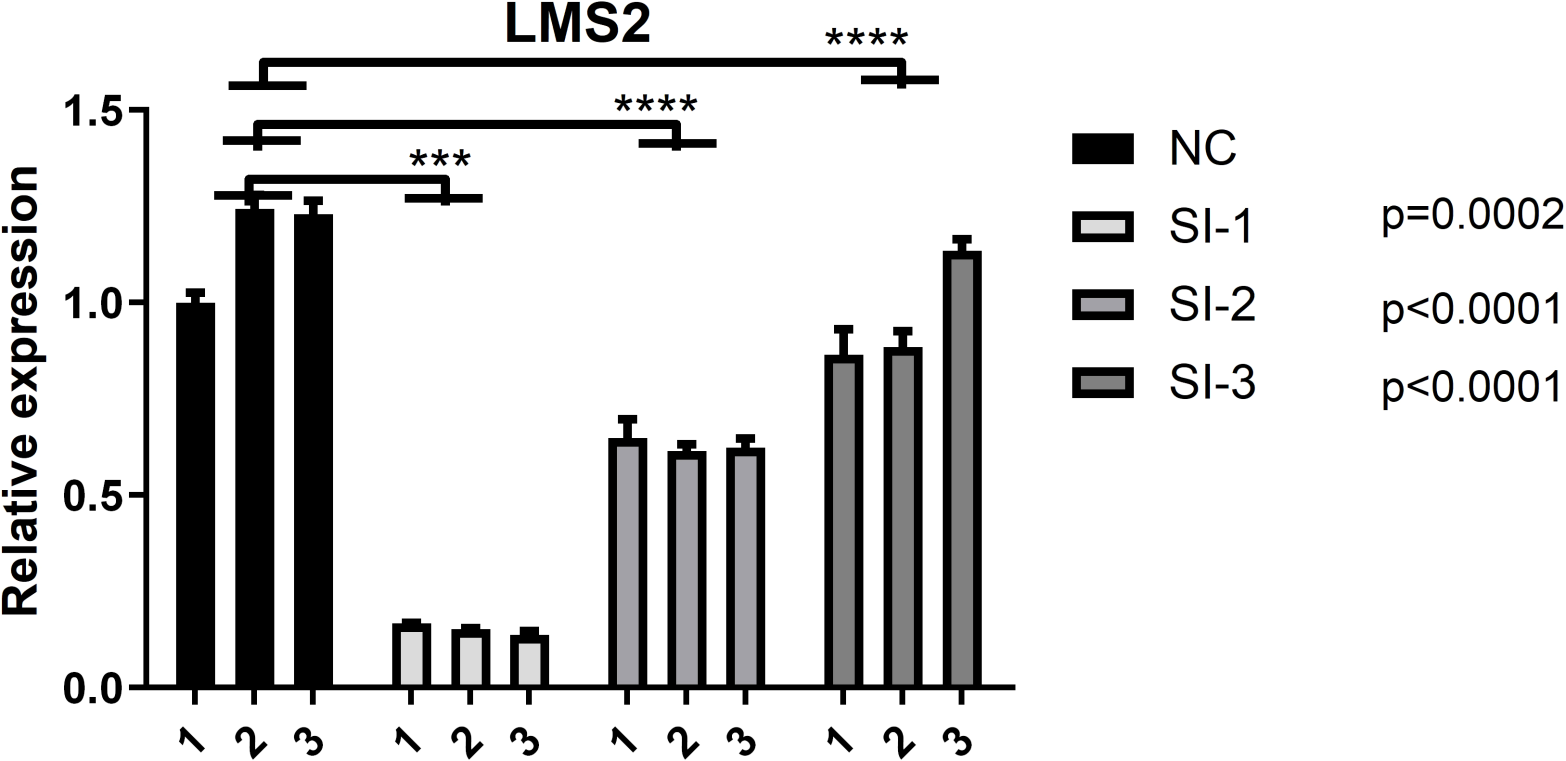
Validation of LMS2 Knockdown Efficiency in T98G Cells. Quantitative PCR (qPCR) analysis of relative LMS2 expression in the negative control (NC) group and three siRNA knockdown groups (SI-1, SI-2, SI-3). All knockdown groups exhibited significantly reduced LMS2 expression compared to the NC group (*p* < 0.0001), with SI-3 demonstrating the highest knockdown efficiency (*p* = 0.0002). Asterisks denote statistical significance (***p < 0.001; ****p < 0.0001).

The human glioblastoma cell line T98G was obtained from the Cell Bank of the Chinese Academy of Sciences (Shanghai, China) and cultured in Dulbecco’s Modified Eagle Medium (DMEM; Procell, PM150210) supplemented with 10% fetal bovine serum (FBS; Hyclone, SH30084.03), 100 U/mL penicillin, and 100 U/mL streptomycin. Cells were maintained at 37°C in a humidified incubator with 5% CO_2_. For *LSM2* knockdown, T98G cells were transfected with small interfering RNA (siRNA) specific to *LSM2* (si*LSM2*) using Lipofectamine RNAiMAX reagent (Invitrogen, 13778150), following the manufacturer’s instructions. Three experimental groups and one control group were established concurrently: the si*LSM2* group and a negative control (NC) group. The efficiency of the transfection was evaluated by RT-qPCR (**Figure 1**), and the harvested cells were preserved for further investigation.

### 2.7 RNA extraction, RNA-seq library construction and sequencing

The glioma cell line T98G was used in this study. Total RNA was extracted from these cells using the QIAGEN RNeasy Kit (Qiagen, Hilden, Germany), following the manufacturer’s protocol. The RNA quality was assessed by measuring the A260/A280 ratio using a SmartSpec Plus spectrophotometer (Bio-Rad, Hercules, CA, USA), with an acceptable ratio of 1.8–2.1. RNA integrity was further confirmed by 1.5% agarose gel electrophoresis. The RNA was treated with RQ1 DNase (Promega, Madison, WI, USA) to remove any genomic DNA contamination. A total of 1 μg of RNA from each sample was used for RNA-seq library preparation.

Polyadenylated mRNAs were enriched using VAHTS mRNA Capture Beads (Vazyme, N401, Nanjing, China). The RNA was then treated with RQ1 DNase (Promega) to remove any residual DNA. Following this, the mRNA was fragmented, and cDNA synthesis was performed using the KAPA Stranded mRNA-Seq Kit for Illumina^®^ Platforms (KK8544, KAPA Biosystems), ensuring the production of strand-specific cDNA. The library preparation involved end repair, A-tailing, and adapter ligation. The strand marked with dUTP (the second cDNA strand) was not amplified, allowing for strand-specific sequencing. The libraries were sequenced on an Illumina NovaSeq 6000 platform using paired-end sequencing (150 nt).

Human GAPDH was used as a control gene to evaluate the effects of LSM2 knockdown (17). The concentration of each transcript was then normalised to the GAPDH mRNA level using the 2^–ΔΔCT method. Primer information is as follows (5′ to 3′): LSM2-Forward: TGACCTGAGCATCTGTGGAA; LSM2-Reverse: CCGCATCCTGTAGCAACTG. Human GAPDH-Forward: GGTCGGAGTCAACGGATTTG; Hum GAPDH-Reverse: GGAAGATGGTGATGGGATTTC (18).

### 2.8 RNA-seq data quality control

The quality of the RNA-seq data was first assessed using FastQC to perform an initial analysis of the sequencing data. Reads were filtered and trimmed to remove adapter sequences and low-quality bases: Reads with a quality score below Q20 were discarded, and only reads with a minimum length of 16 bases were retained. The first 3 bases from each read were clipped to remove potential sequencing bias. Effective reads, known as clean reads, were extracted using the following criteria: Removal of adapter sequences and low-quality bases (Q20 threshold). Reads were trimmed to a length of at least 16 bases. Clean reads were mapped to the human reference genome (GRCh38) using HISAT2 alignment tool. The following mapping steps were performed: 1) Total mapped reads were computed, with reads that could be uniquely mapped to a single location on the genome categorised as “uniquely mapped”. 2) Reads that mapped to multiple locations were classified as “multiple mapped” reads, typically representing rRNA and tRNA sequences. For subsequent analyses, only “uniquely mapped” reads were used to ensure the accuracy and reliability of the results.

### 2.9 Differential Gene Expression analysis

Gene expression levels were quantified using HTSeq and normalised using the DESeq2 package in R. Subsequently, rigorous statistical analysis was performed using the DESeq2 software package (19, 20). DESeq2 model raw read counts by applying scaling factors to normalise library size differences, followed by estimation and shrinkage of gene dispersion parameters to refine the negative binomial distribution model. Hypothesis testing was carried out using either the Wald test or likelihood ratio test, and results were adjusted for multiple comparisons using the Benjamini-Hochberg false discovery rate (FDR) method. DEGs were defined as genes meeting the following criteria: an absolute fold change (FC) ratio of ≥2 or ≤0.5 and statistical significance with an FDR-adjusted p-value <0.05. Significant DEGs were prioritised based on both biological relevance (FC threshold) and statistical confidence (FDR correction), ensuring robust identification of genes with differential expression across experimental conditions.

### 2.10 Alternative Splicing analysis

AS is a critical post-transcriptional regulatory mechanism in gene expression, allowing a single gene to generate multiple mRNA transcripts by selectively including or excluding different exons or introns. This genetic versatility facilitates the production of diverse proteins, thereby increasing the complexity of the genome (21).

To detect alternative splicing events, the splice junctions (SJs) across all samples were identified using the HISAT2 alignment tool. Alternative splicing events were identified using the ABLas program, which performs comprehensive analysis and classification of AS events based on the detected splice junctions. The AS events were categorised into several types, including Exon Skipping (ES), Alternative 5’ Splice Site (A5SS), Alternative 3’ Splice Site (A3SS), Intron Retention (IntronR), and Mutually Exclusive Exons (MXE), among others (22). These event types were classified using previously described criteria. Each AS event type was then quantified across all samples, and the proportion of each event type relative to the total number of splice junctions detected was calculated. Splicing event significance was assessed with a threshold of p < 0.05.

### 2.11 Functional enrichment analysis

The Gene Ontology (GO) database classifies genes and proteins into three functional categories: molecular function, biological process, and cellular component. To perform enrichment analysis, DEGs, differentially spliced genes (RASG), and genes that overlap between DEGs and RASG were mapped to GO terms. To minimise false positives, a hypergeometric test was employed, followed by Benjamini-Hochberg FDR correction for multiple comparisons. Significantly enriched terms were defined as those with an adjusted p-value of < 0.05, and the top ten terms, ranked by significance, were selected for detailed presentation.

The Kyoto Encyclopedia of Genes and Genomes (KEGG) integrates large-scale molecular datasets derived from genome sequencing and high-throughput experimental techniques. Pathway enrichment analysis was conducted to assess the potential functional implications of the differentially expressed and spliced genes. Genes were mapped to KEGG pathways, and their significance was evaluated using a hypergeometric distribution test. Multiple testing correction was performed using the Benjamini-Hochberg method, with an FDR-adjusted p-value of < 0.05. The top ten enriched pathways, ranked by their adjusted p-value, were selected for further investigation.

### 2.12 Protein-Protein Interaction network and core gene screening

To identify key hub genes, a PPI network was constructed using the STRING database (https://cn.string-db.org/) (23). Genes with a confidence score >0.4 were selected, and the PPI network was analysed using Cytoscape (version 3.7.2) and the MCODE plugin to identify significant subnetworks. Hub genes were further analysed for prognostic significance using Kaplan-Meier survival analysis.

### 2.13 Statistical analysis

DEGs were identified using DESeq2 in R, with raw count data normalised via its default method. Differential expression was determined by the Wald test (FDR-adjusted p < 0.05). Alternative splicing events were analysed by comparing splicing levels across conditions using a t-test (p < 0.05 for significance).

Functional enrichment analysis of DEGs and RASGs employed hypergeometric testing, with GO and KEGG pathway analyses assessed using FDR-adjusted p < 0.05. The Benjamini-Hochberg method was applied for multiple testing correction throughout.

All analyses used R v4.0.2, with statistical significance set at p < 0.05 unless stated.

## 3 Results

### 3.1 *LSM2* expression in gliomas and its prognostic significance

**Figure 2:**
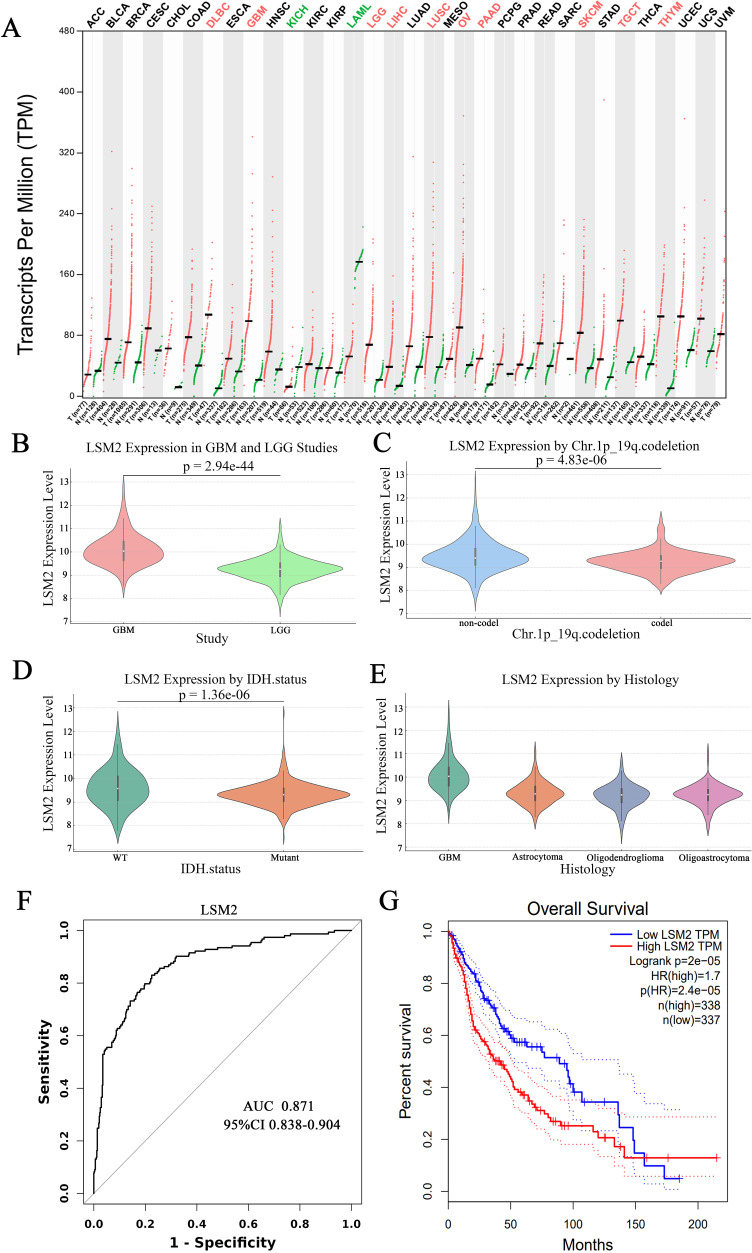
Comparison of *LSM2* expression levels in different glioma types and genetic markers. **(A)** *LSM2* pan-cancer analysis; **(B)** Differences in *LSM2* expression in GBM and LGG; **(C)** Relationship between *LSM2* expression and chromosome 1p/19q co-deletion status; **(D)** Relationship between *LSM2* expression and IDH1 mutation status; **(E)** Expression of *LSM2* in different glioma pathological types; **(F)** ROC curves of *LSM2* differentiating GBM from LGG; **(G)** Kaplan-Meier survival curves of the effect of *LSM2* expression level on patients’ overall survival. p < 0.05 is statistically significant.

A pan-cancer analysis of LSM2 expression using the GEPIA database revealed significantly higher expression of LSM2 in tumour samples compared to normal samples across various cancer types, including glioblastoma (GBM) and lower-grade glioma (LGG) (p < 0.05) (**Figure 2A**). Specifically, LSM2 expression was significantly elevated in GBM (163 cases) compared to LGG (518 cases) (**Figure 2B**). Furthermore, LSM2 expression was significantly higher in gliomas with 1p/19q non-deletion (494 cases) than in those with 1p/19q co-deletion (169 cases) (p < 0.001), as well as in IDH1-mutant gliomas (429 cases) compared to IDH1 wild-type cases (233 cases) (p < 0.001) (**Figures 2C, D**).

Subgroup analysis by glioma histological type demonstrated higher LSM2 expression in GBM compared to astrocytomas, oligodendrogliomas, and mixed gliomas (**Figure 2E**). The diagnostic potential of LSM2 was assessed using receiver operating characteristic (ROC) curve analysis, which yielded an area under the curve (AUC) of 0.871 (95% CI: 0.838–0.904), confirming that LSM2 is a robust marker for distinguishing GBM from LGG (**Figure 2F**). Kaplan-Meier survival analysis demonstrated that patients with high LSM2 expression had significantly shorter overall survival compared to those with low LSM2 expression (HR = 1.7, 95% CI = 1.31–2.20, p < 0.001) (**Figure 2G**).

### 3.2 Immune cell infiltration and LSM2 expression

**Figure 3:**
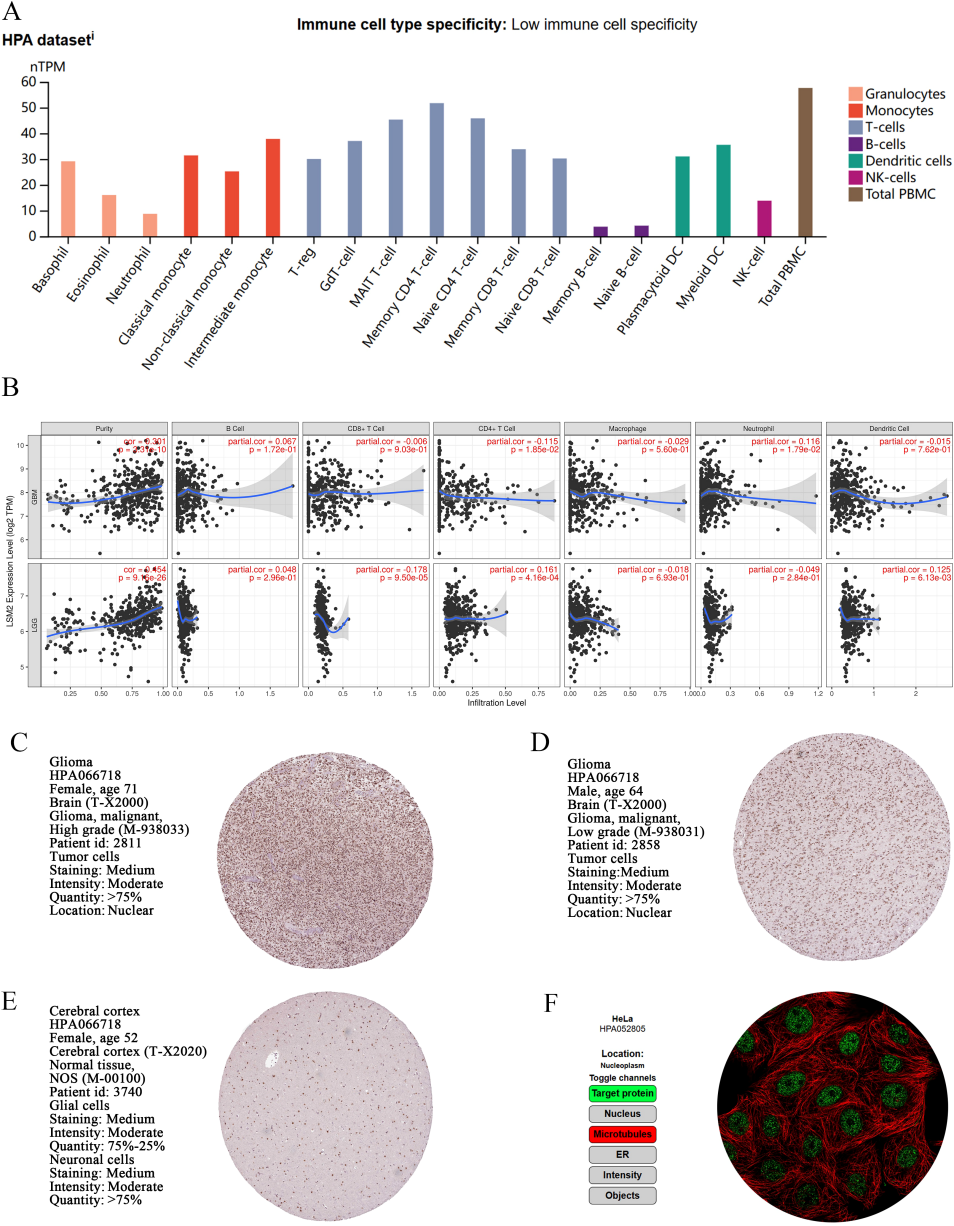
Expression and localisation of *LSM2* in immune cells, glioma tissue and normal brain tissue. **(A)** nTPM levels of *LSM2* in immune cells. **(B)** Analysis of the correlation between immune cell infiltration and *LSM2* expression in gliomas. **(C-E)** Immunohistochemical analysis of *LSM2* in GBM, LGG and normal brain tissue. **(F)** Immunofluorescence analysis of HeLa glioma cells.

We analysed the normalised RNA expression (nTPM) levels of LSM2 in 18 immune cell types and total peripheral blood mononuclear cells (PBMCs) using data from the HPA database. The results indicated that LSM2 is expressed across a wide variety of immune cell types, with particularly high expression levels observed in total PBMCs, non-classical monocytes, intermediate monocytes, and early CD4+ T cells (**Figure 3A**). We analysed the relationship between LSM2 expression and immune cell infiltration levels using the TIMER platform. In GBM, LSM2 expression exhibited a significant positive correlation with tumour purity (r = 0.301, p < 0.001) and neutrophil infiltration (r = 0.116, p < 0.05), and a weak negative correlation with CD4+ T cell infiltration (r = -0.115, p = 1.85e-02). In contrast, in LGG, LSM2 expression positively correlated with tumour purity (r = 0.154, p < 0.001) and CD4+ T cell infiltration (r = 0.161, p < 0.001), and negatively correlated with CD8+ T cell infiltration (r = -0.178, p < 0.001) (**Figure 3B**).

Immunohistochemical analysis of both high-grade and low-grade glioma tissues revealed strong nuclear localisation of LSM2, with moderate staining intensity (**Figures 3C, D**). This expression pattern suggests that LSM2 may play a key role in the proliferation, differentiation, or other nuclear functions of tumour cells. Additionally, LSM2 exhibited extensive nuclear expression in normal brain tissue, indicating its importance in the physiological functions of the nervous system (**Figure 3E**). Further immunofluorescence analysis of HeLa cells demonstrated that LSM2 is primarily localised to the nucleolus (**Figure 3F**).

### 3.3 Genetic alterations of LSM2 in gliomas

**Figure 4:**
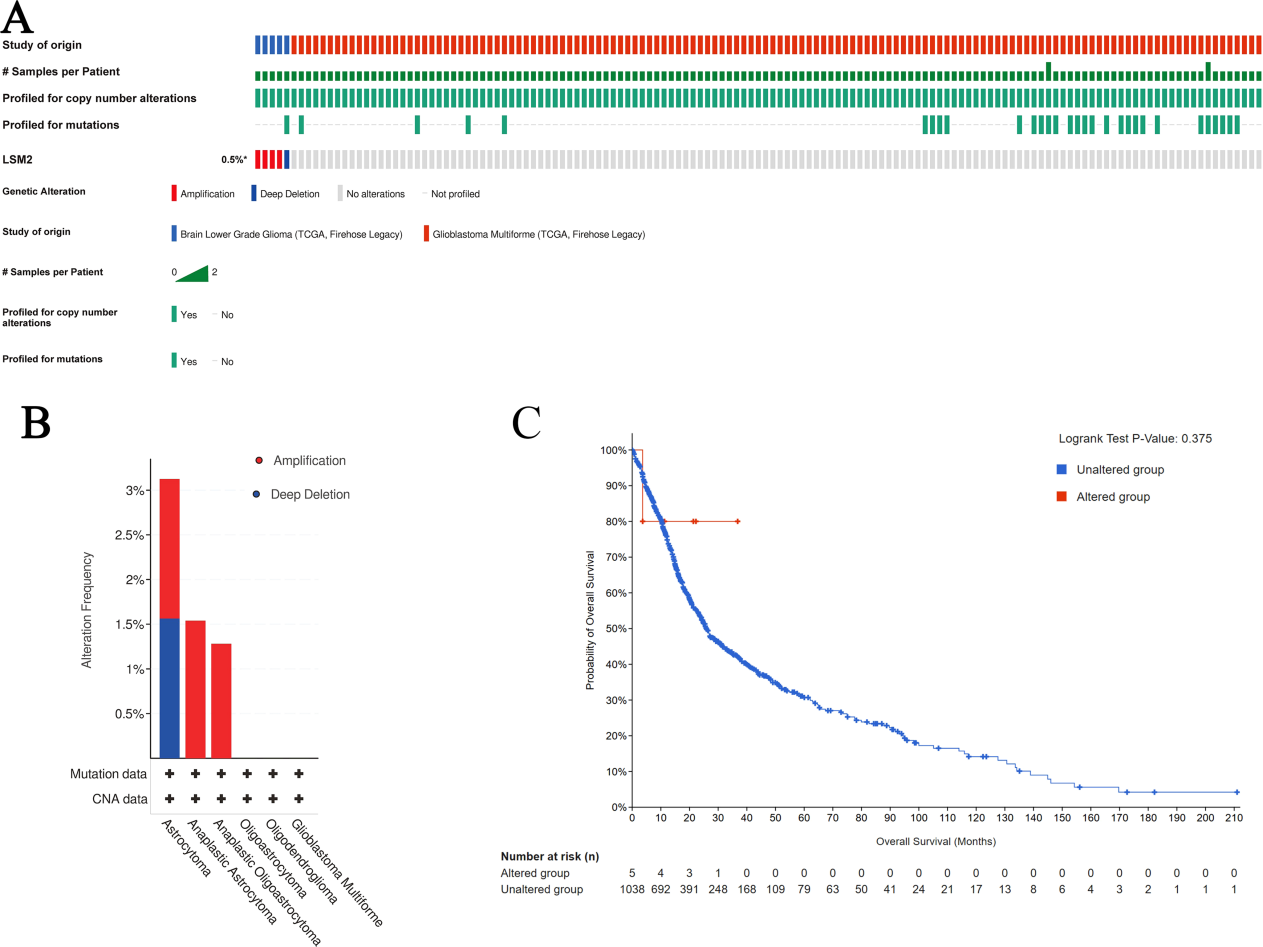
Results of LSM2 gene mutation and survival analysis in gliomas. **(A)** OncoPrint visual summary of LSM2 gene alterations. **(B)** Overview of LSM family gene alterations in gliomas. **(C)** Kaplan - Meier plot comparing OS in patients with LSM2 mutations in gliomas to patients without LSM2 mutations (p = 0.375). p < 0.05 statistically significant.

Analysis of TCGA data via cBioPortal revealed a low mutation frequency of LSM2 in gliomas (0% in LGG, 5% in GBM, predominantly amplifications/deletions) (**Figures 4A, B**). Kaplan-Meier survival analysis showed no significant association between LSM2 alterations and patient survival (p=0.375, **Figure 4C**), suggesting that genetic alterations may not be the primary driver of LSM2’s role in glioma progression.

### 3.4 Differential gene expression and enrichment analysis after LSM2 knockdown

**Figure 5:**
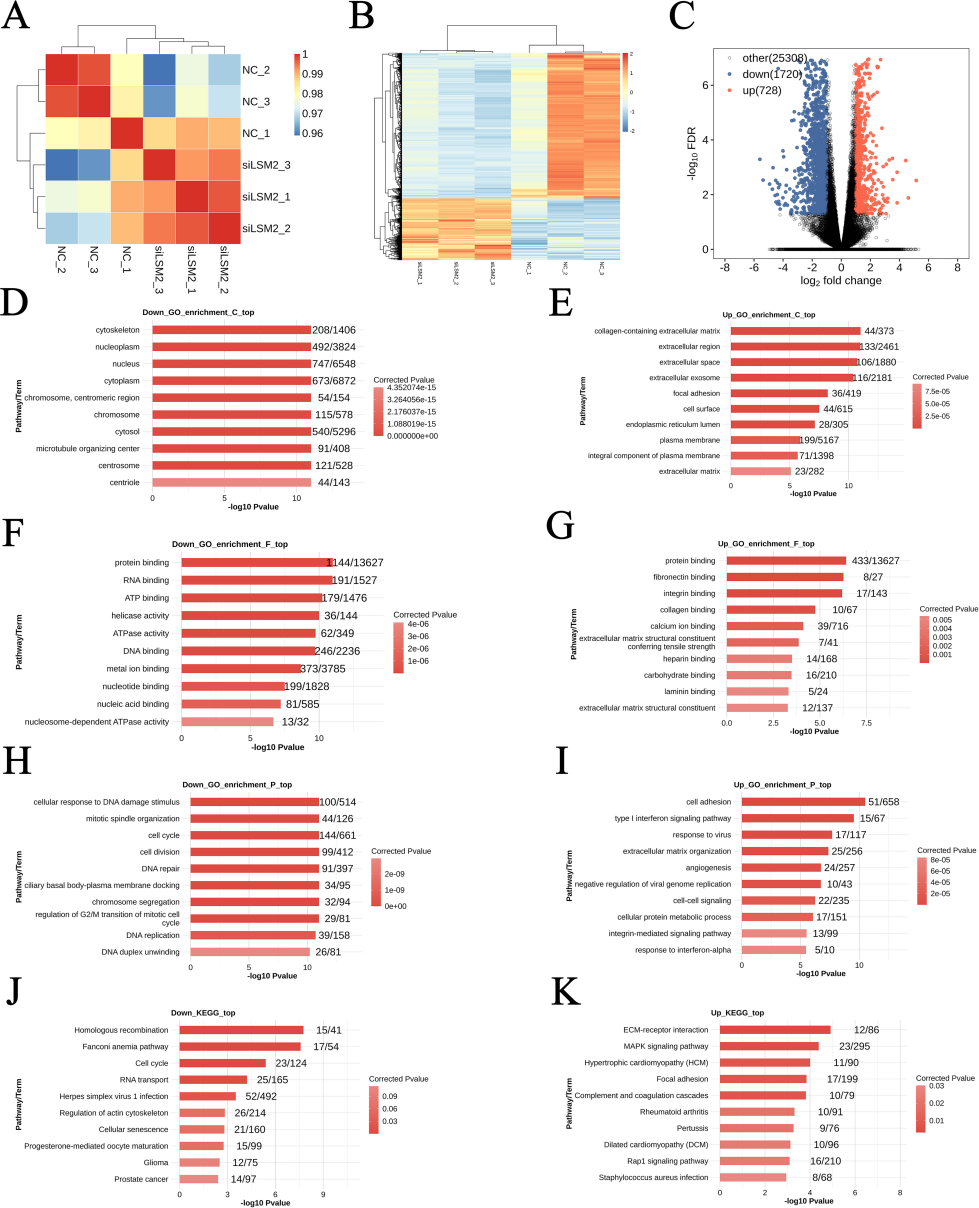
Differential gene analysis and GO and KEGG enrichment analysis of T98G cell lines after *LSM2* knockdown. **(A)** Sample clustering analysis; **(B)** Differential gene clustering heat map; **(C)** DEG volcano plot; **(D–I)** Three categories of GO enrichment analysis: molecular function, biological process, and cellular component of differentially ex-pressed genes; **(J, K)** KEGG pathway enrichment analysis of differentially expressed genes. p < 0.05 was considered statistically significant.

In the T98G cell line, significant changes in gene expression were observed between the experimental group (siLSM2, n = 3) and the control group (NC, n = 3) (**Figure 5A**). Comparative analysis of gene expression profiles using the DESeq tool identified 728 genes with significantly increased expression and 1,720 genes with significantly decreased expression in LSM2 knockdown cells compared to the control group (**Figure 5C**). These findings suggest that LSM2 plays a crucial role in regulating various biological processes and signalling pathways. Heatmap clustering analysis further confirmed distinct differences in expression patterns between the two groups, indicating that LSM2 knockdown induces widespread transcriptional changes within the cells (**Figure 5B**).

GO and KEGG pathway enrichment analyses were performed on the differentially expressed genes (DEGs) to investigate the underlying molecular mechanisms. GO analysis revealed that upregulated DEGs were significantly enriched in the collagen-containing extracellular matrix (44 genes, p < 0.05) and the extracellular region (133 genes, p < 0.05), suggesting that LSM2 knockdown may affect cell-matrix interactions (**Figure 5E**). At the molecular function level, upregulated genes were predominantly involved in protein binding (433 genes, p < 0.05) and fibronectin binding (8 genes, p < 0.05), suggesting that LSM2 may regulate molecular interactions related to cell adhesion and signalling (**Figure 5G**). In terms of biological processes, significantly enriched pathways included cell adhesion (51 genes, p < 0.05) and the type I interferon signalling pathway (15 genes, p < 0.05), implying that LSM2 knockdown may enhance cell adhesion and activate antiviral responses (**Figure 5I**).

In contrast, downregulated DEGs were enriched in the cytoskeleton (208 genes, p < 0.05) and nucleoplasm (492 genes, p < 0.05), indicating that LSM2 knockdown significantly affects cellular structure and nuclear function (**Figure 5D**). At the molecular function level, downregulated genes were significantly associated with protein binding (1,144 genes, p < 0.05) and RNA binding (191 genes, p < 0.05), highlighting that LSM2 plays a key role in regulating protein interactions and RNA homeostasis (**Figure 5F**). Enriched biological processes included the cellular response to DNA damage (100 genes, p < 0.05) and mitotic spindle organisation (44 genes, p < 0.05), suggesting that LSM2 knockdown may impair genomic damage repair and interfere with normal cell division (**Figure 5H**).

KEGG pathway analysis revealed that upregulated DEGs were closely associated with ECM-receptor interaction (12 genes, p < 0.05) and the MAPK signalling pathway (23 genes, p < 0.05), suggesting that LSM2 knockdown may regulate extracellular matrix interactions and signalling mechanisms (**Figure 5K**). Downregulated DEGs were enriched in homologous recombination (15 genes, p < 0.05) and the Fanconi anaemia pathway (17 genes, p < 0.05), indicating that LSM2 knockdown may affect DNA repair and genome stability (**Figure 5J**).

### 3.5 Alternative splicing events induced by LSM2 knockdown

We analysed LSM2-regulated alternative splicing events (RASEs). A t-test with a threshold of p < 0.05 was used to identify significant AS events. A total of 858 upregulated AS events were identified, with the highest frequency observed in A5SS (189 events), followed by A3SS (187 events). Conversely, AS analysis detected 1,091 significantly downregulated splicing events, with A3SS (257 events) and A5SS (256 events) as the most common types (p < 0.05).

#### 3.5.1 GO enrichment analysis of AS events

**Figure 6:**
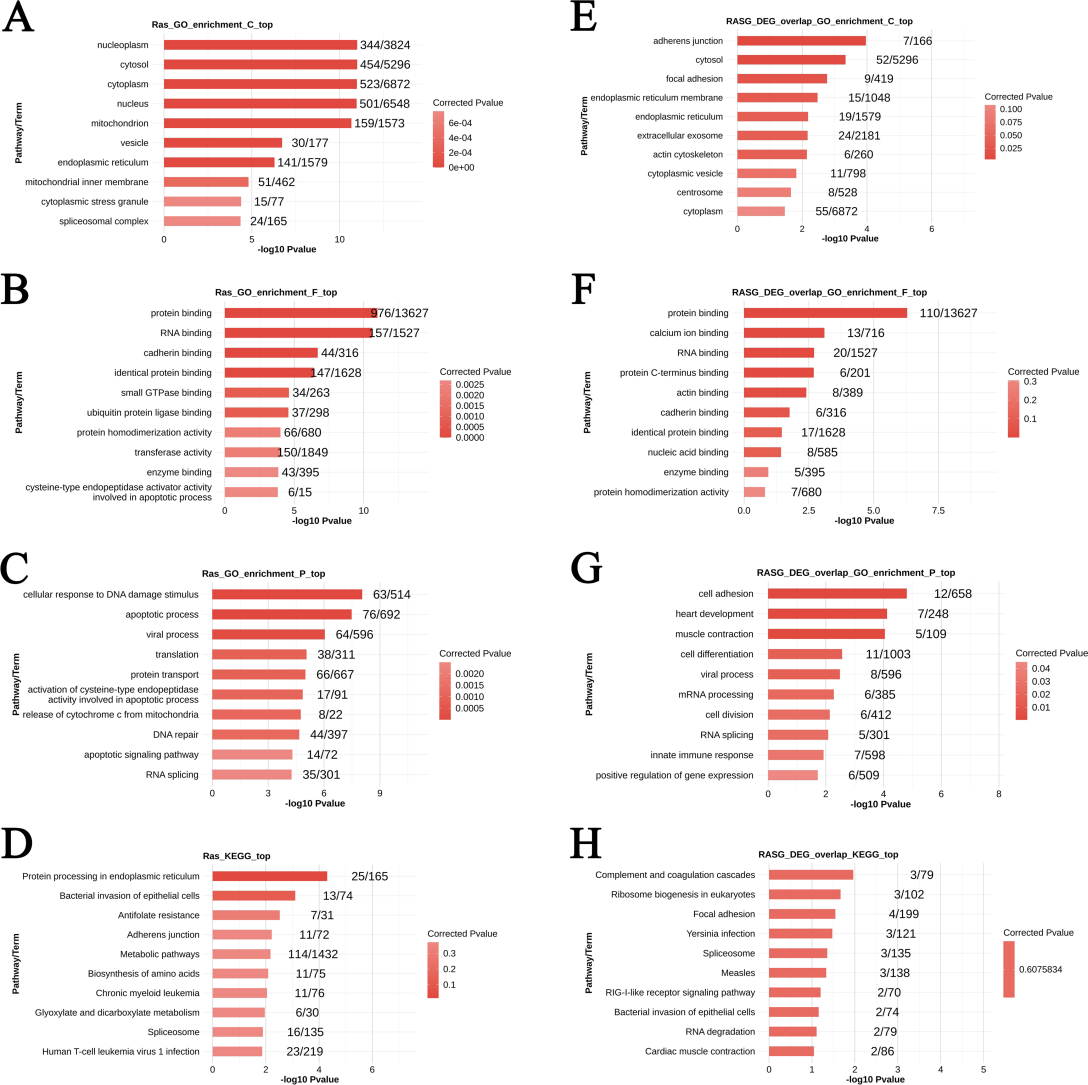
Differential alternative splicing genes and overlapping genes for GO enrichment versus KEGG pathway enrichment. p < 0.05 was considered statistically significant. **(A–C)** GO enrichment analysis of differentially spliced genes. **(D)** KEGG pathway enrichment analysis of differentially spliced genes. **(E–G)** GO enrichment analysis of overlapping genes. **(H)** KEGG pathway enrichment analysis of overlapping genes.

GO analysis of LSM2-RASGs (genes associated with RASEs) revealed significant enrichment in cellular components such as the nucleoplasm (344 genes, p < 0.05), cytosol (454 genes, p < 0.05), and endoplasmic reticulum (141 genes, p < 0.05). These findings suggest involvement in intracellular organisation, particularly nucleoplasmic functions and processes associated with the endoplasmic reticulum. Enrichment in vesicles (30 genes, p < 0.05) and cytoplasmic stress granules (15 genes, p < 0.05) indicates roles in intracellular transport and regulation of stress responses (**Figure 6A**).

At the molecular function level, LSM2-RASGs were enriched in protein binding (976 genes, p < 0.05), RNA binding (157 genes, p < 0.05), and cadherin binding (44 genes, p < 0.05), suggesting they mediate protein-protein and protein-RNA interactions. These results highlight their potential roles in cell adhesion and RNA-related processes (**Figure 6B**).

In terms of biological processes, LSM2-RASGs were enriched in pathways such as the cellular response to DNA damage (63 genes, p < 0.05), apoptotic processes (76 genes, p < 0.05), and the viral process (64 genes, p < 0.05). Enrichment in DNA repair (44 genes, p < 0.05) and RNA splicing (35 genes, p < 0.05) further emphasises their roles in maintaining genomic stability and regulating post-transcriptional processes (**Figure 6C**).

#### 3.5.2 KEGG pathway analysis of AS events

KEGG analysis revealed that LSM2-RASGs were significantly enriched in pathways such as protein processing in the endoplasmic reticulum (25 genes, p < 0.05) and bacterial invasion of epithelial cells (13 genes, p < 0.05). These findings suggest that LSM2 knockdown may disrupt protein folding and modification processes, leading to impaired cellular secretion and stress responses (**Figure 6D**).

#### 3.5.3 Integration of RASEs and DEGs

Integration analysis of RASEs and DEGs identified 139 overlapping genes significantly altered at both the expression and splicing levels. Enrichment analysis of these genes revealed significant roles in cellular structure, extracellular signalling, and molecular interactions.

For cellular components, enrichment analysis showed involvement in adherens junctions (7/166 genes, p < 0.05), the cytosol (52/5,296 genes, p < 0.05), and the endoplasmic reticulum membrane (15/1,048 genes, p < 0.05). Additional enrichment in extracellular exosomes (24/2,181 genes, corrected p < 0.05) and cytoplasmic vesicles (11/798 genes, corrected p < 0.05) suggests contributions to intracellular transport and extracellular communication (**Figure 6E**).

At the molecular function level, overlapping genes were enriched in protein binding (110/13,627 genes, p < 0.05), calcium ion binding (20/1,527 genes, p < 0.1), and RNA binding (6/201 genes, p < 0.05), highlighting their roles in protein interactions, calcium signalling, and RNA regulation (**Figure 6F**).

In terms of biological processes, overlapping genes were associated with cell adhesion (12/658 genes, p < 0.05), heart development (7/248 genes, p < 0.05), and muscle contraction (5/109 genes, p < 0.05). These findings suggest their involvement in tissue remodelling, cellular migration, and contractile processes (**Figure 6G**).

KEGG analysis of overlapping genes revealed significant enrichment in pathways such as complement and coagulation cascades (3 genes, p < 0.05) and ribosome biogenesis in eukaryotes (3 genes, p < 0.05). These results suggest that LSM2 knockdown may interfere with immune responses, coagulation, and ribosome production, ultimately impacting cellular growth, proliferation, and metabolism (**Figure 6H**).

### 3.6 Protein-protein interaction network and core gene identification

**Figure 7:**
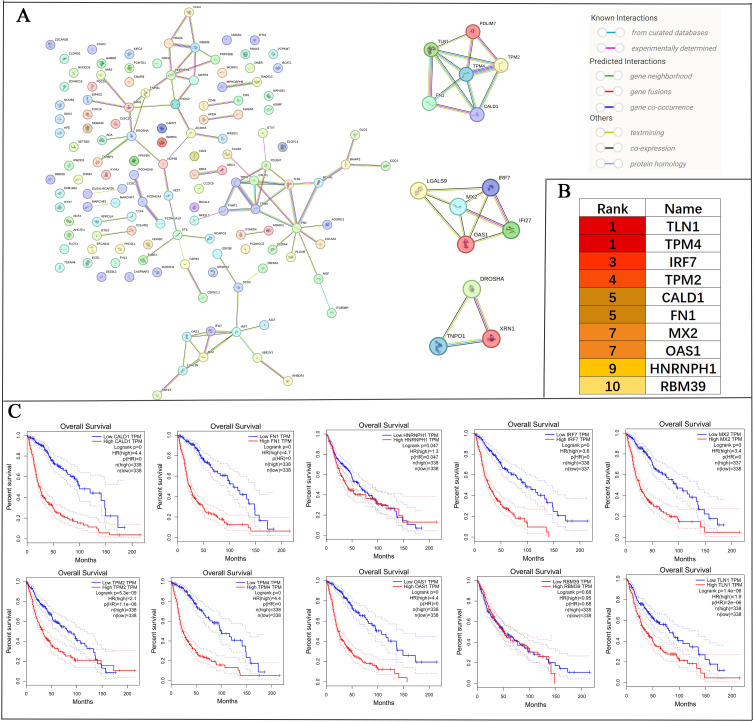
PPI network analysis of 139 alternative splicing-regulated and differentially expressed overlapping genes, and screening and prognostic analysis of core genes. **(A)** The PPI network was constructed using the STRING database and subdivided into three prominent subnetworks with Cytoscape software. **(B)** The top 10 core genes in the PPI network were identified using the MCC topology analysis method via the CytoHubba plug-in in Cytoscape. **(C)** The prognostic impact of these 10 core genes in glioma patients was evaluated using Kaplan-Meier survival curves. p < 0.05 was considered statistically significant.

The PPI network of DEGs and ASEs identified significant interactions among 139 overlapping genes. The network was further divided into three prominent subnetworks using the MCODE plug-in in Cytoscape software (**Figure 7A**).

To identify key genes within the PPI network, the CytoHubba plug-in in Cytoscape was utilised to perform MCC topology analysis, resulting in the selection of the top ten hub genes: TLN1, TPM4, IRF7, TPM2, CALD1, FN1, MX2, OAS1, HNRNPH1, and RBM39 (**Figure 7B**). Kaplan-Meier survival curve analysis demonstrated that elevated expression of TLN1, TPM4, IRF7, TPM2, CALD1, FN1, MX2, OAS1, HNRNPH1, and RBM39 was significantly associated with reduced overall survival (p < 0.05) (**Figure 7C**).

## 4 Discussion

Glioma, particularly glioblastoma (GBM), remains one of the most challenging cancers to treat, despite advances in multimodal therapies, including surgery, radiotherapy, and chemotherapy (3). LSM2 - a core component of the LSM family - may drive malignant phenotypes through regulation of pivotal RNA metabolic pathways (9). This investigation employed an integrated multidimensional approach, incorporating bioinformatics prediction, gene knockout models and functional enrichment analysis, to systematically elucidate LSM2’s central regulatory role in glioma progression. Our study highlights LSM2 as a critical player in glioma progression, particularly through its regulation of RNA splicing and its impact on key pathways involved in tumour biology, such as cell adhesion, DNA repair, and immune modulation.

Previous studies have reported that RBPs like LSM2 are involved in the regulation of mRNA stability, splicing, and translation, all of which are crucial for tumour progression. LSM2 has been shown to modulate critical cellular processes, including RNA splicing, which is essential for maintaining cellular homeostasis. Our results corroborate these findings, demonstrating that LSM2 expression is significantly upregulated in gliomas, especially in GBM, and correlates with poor prognosis. This is consistent with previous studies linking elevated expression of LSM2 to malignancy in other cancers, such as breast cancer and hepatocellular carcinoma.

### 4.1 LSM2’s impact on glioma cell biology and tumour progression

Our investigation revealed elevated LSM2 expression in gliomas through exploration of the TCGA database, LSM2 expression is significantly higher in GBM compared to lower-grade gliomas (LGG), and its expression correlates with the presence of IDH1 mutations and the 1p/19q non-deletion status. These findings suggest that LSM2 may contribute to the aggressiveness of gliomas, particularly in more malignant subtypes such as GBM. This is further supported by our survival analysis, where high LSM2 expression was associated with shorter overall survival in glioma patients. These results imply that LSM2 could serve as a prognostic biomarker for glioma, particularly in distinguishing GBM from LGG and predicting patient outcomes. While previous TCGA-based studies have identified candidate glioma markers such as SCN3B and CDK2 (24, 25), our findings propose LSM2 as a novel candidate marker.

Our findings demonstrate that LSM2 plays a crucial role in glioma progression through its influence on various cellular pathways, such as the cell cycle, DNA repair, RNA splicing, and cell adhesion. Knockdown of LSM2 significantly impacted glioma cell behaviour, with alterations observed in critical pathways that regulate tumour proliferation, survival, and invasiveness. Specifically, LSM2 depletion resulted in the upregulation of extracellular matrix (ECM) genes like FN1 and COL1A1, which could disrupt tumour cell-ECM interactions and potentially diminish the invasive phenotypes of glioma cells. This suggests that LSM2’s regulatory role in RNA splicing affects adhesion molecules, which in turn influences tumour cell migration and invasion.

These findings support the established role of the LSM protein family in maintaining RNA stability and regulating post-transcriptional processes (26). By modulating splicing events, LSM2 may help maintain the integrity of cytoskeletal structures and nuclear functions that are critical for tumour progression. The regulation of RNA metabolism by LSM2 further connects these findings to previous research showing the importance of RNA processing in malignancy.

Moreover, our data show that LSM2 depletion leads to significant changes in RNA splicing, specifically with the predominance of alternative 5’ (A5SS) and 3’ (A3SS) splice site events. These splicing alterations are associated with key pathways such as the spliceosome, DNA repair, and ECM-receptor interactions. Given the importance of splicing in tumour progression, these findings emphasise LSM2’s pivotal role in driving glioma progression through splicing regulation.

### 4.2 Splicing dysregulation and tumour progression

Consistent with previous studies, our results suggest that dysregulation of RNA splicing plays a critical role in tumourigenesis by generating aberrant splice variants of key oncogenes (27). We observed that LSM2 regulates several splicing events that influence genes involved in critical processes such as cell cycle progression, DNA repair, and immune modulation. Disruption of these pathways following LSM2 knockdown likely impairs glioma cell proliferation and makes the tumour cells more vulnerable to environmental stress. This finding highlights the potential therapeutic benefit of targeting LSM2 to impair glioma progression by modulating these splicing events.

Moreover, integrated Gene Ontology (GO) and Kyoto Encyclopaedia of Genes and Genomes (KEGG) pathway analyses of overlapping genes revealed that LSM2’s influence extends beyond splicing. LSM2 appears to regulate cytoskeletal stability, protein translation, and cell-matrix interactions. Pathway enrichment in adherens junctions, focal adhesions, and complement cascades suggests that LSM2 affects tumour cell migration and invasion by influencing interactions with the tumour microenvironment. These findings further demonstrate how LSM2 integrates various cellular pathways to sustain tumour aggressiveness and highlight its potential as a target for therapeutic intervention.

### 4.3 Key genes and potential therapeutic targets

Our analysis identified 139 overlapping genes. Through protein-protein interaction (PPI) network analysis, these genes were grouped into three major sub-networks: cytoskeletal remodelling and adhesion, immune modulation and antiviral response, and RNA metabolism and post-transcriptional regulation. Using MCC topology analysis in the CytoHubba plugin, we identified 10 key DEGs involved in glioma progression. Of these, eight genes (TLN1, TPM4, TPM2, CALD1, FN1, IRF7, MX2, and OAS1) were significantly correlated with reduced overall survival. These hub genes —key regulators of cytoskeletal dynamics and cell migration—implies that LSM2 may promote glioma progression by dysregulating these effector molecules, potentially via aberrant splicing of their pre-mRNAs. CALD1 has been shown to regulate glioma progression by promoting tumour angiogenesis (28). The upregulation of TPM4 predicts shorter survival time in glioma patients and plays a crucial role in the pro-EMT process through synergistic interactions with the pro-EMT signalling pathway and key molecules (29). TLN1 and TPM2 are key regulators of cytoskeletal dynamics and cell-matrix interactions, directly promoting glioma cell migration and invasion (30). FN1 promotes the migration and invasion of GBM cells and is a key candidate for mediating the function of cancer-associated fibroblasts (31). Meanwhile, IRF7, MX2, and OAS1 are key mediators in immune regulatory pathways, and their overexpression may facilitate immune evasion in gliomas.

### 4.4 LSM2 and the immune microenvironment

In this study, we observed that LSM2 expression is closely associated with immune cell infiltration levels in gliomas. Specifically, in glioblastoma (GBM), LSM2 expression was positively correlated with tumour purity and neutrophil infiltration, but negatively correlated with CD4+ T cell infiltration. In contrast, in lower-grade gliomas (LGG), LSM2 expression showed a positive correlation with tumour purity and CD4+ T cell infiltration, but a negative correlation with CD8+ T cell infiltration.

These findings suggest that LSM2 may play a role in modulating the tumour immune microenvironment, potentially influencing immune cell function or tumour-associated immune responses. In GBM, LSM2 could promote neutrophil infiltration while inhibiting CD4+ T cell infiltration, which may facilitate immune evasion by the tumour. On the other hand, in LGG, LSM2 may affect immune surveillance by promoting CD4+ T cell infiltration while inhibiting CD8+ T cell infiltration. This dual role in immune modulation could contribute to the differences observed between high- and low-grade gliomas in terms of immune response and tumour progression.

It is important to acknowledge that these results are primarily based on bioinformatics analysis, and further experimental and clinical studies are needed to validate these observations. Future studies employing single-cell RNA sequencing and *in vivo* models are warranted to elucidate the precise mechanisms by which LSM2 modulates immune responses and to assess the therapeutic potential of targeting LSM2 to restore effective antitumour immunity (32). In addition, in combination with the iMLGAM scoring system, the potential role of LSM2 in immunotherapy can be more comprehensively assessed, providing important clues for the development of new therapeutic strategies (33).

### 4.5 Therapeutic implications and future directions

The significant role of LSM2 in glioma progression presents several promising therapeutic opportunities. Targeting LSM2 could potentially disrupt its regulation of splicing and related pathways, leading to reduced tumour cell proliferation, invasion, and immune evasion. Moreover, the identification of key genes regulated by LSM2 provides potential biomarkers for glioma prognosis, which could help in developing diagnostic and therapeutic strategies. Liquid biopsy techniques, such as circulating tumour DNA (ctDNA) analysis, could be explored to monitor LSM2 expression and its molecular network, further enhancing diagnostic capabilities for glioma (34, 35).

However, several limitations need to be addressed in future research. Our study primarily relied on bioinformatics analyses and *in vitro* experiments, and thus, *in vivo* validation in animal models is needed to confirm the therapeutic potential of LSM2-targeted therapies. Additionally, the mechanisms by which LSM2 influences immune responses within the tumour microenvironment remain unclear and should be investigated further. Understanding how LSM2 interacts with immune cells and modulates their activity could open new avenues for glioma treatment, particularly in combination with immunotherapy.

## 5 Conclusions

This study establishes LSM2 as a central player in glioma progression, affecting multiple cellular processes such as RNA splicing, cell adhesion, and immune modulation. LSM2’s role in regulating these pathways highlights its potential as both a prognostic biomarker and a therapeutic target for gliomas. Future studies should focus on further exploring the precise mechanisms by which LSM2 influences tumour behaviour and immune responses, with the ultimate goal of developing effective therapies for glioma patients.

## Data Availability

The data supporting the conclusions of this article are derived from both publicly available databases and original experimental research. Specifically, clinical and transcriptomic data were obtained from TCGA (https://portal.gdc.cancer.gov/), GEPIA (http://gepia.cancer-pku.cn/), cBioPortal (https://www.cbioportal.org/), TIMER (https://cistrome.shinyapps.io/timer/) and HPA (https://www.proteinatlas.org/). Additionally, the datasets generated and analysed during the current study are available in the NCBI Sequence Read Archive (SRA) repository, under the project number PRJNA1209206, and can be accessed via the following link: https://dataview.ncbi.nlm.nih.gov/object/PRJNA1209206?reviewer=ph9a89t9iglrrpb4ia2pk4gsab.

## References

[1] Bray F Laversanne M Sung H Ferlay J Siegel RL Soerjomataram I. Global cancer statistics 2022: GLOBOCAN estimates of incidence and mortality worldwide for 36 cancers in 185 countries. CA Cancer J Clin. (2024) 74:229–63. doi: 10.3322/caac.21834 38572751

[2] Sonkin D Thomas A Teicher BA. Cancer treatments: Past, present, and future. Cancer Genet. (2024) 286–287:18–24. doi: 10.1016/j.cancergen.2024.06.002 PMC11338712 38909530

[3] Tan AC Ashley DM López GY Malinzak M Friedman HS Khasraw M. Management of glioblastoma: State of the art and future directions. CA Cancer J Clin. (2020) 70:299–312. doi: 10.3322/caac.21613 32478924

[4] Goodwin S McPherson JD McCombie WR. Coming of age: ten years of next-generation sequencing technologies. Nat Rev Genet. (2016) 17:333–51. doi: 10.1038/nrg.2016.49 PMC10373632 27184599

[5] Anand U Dey A Chandel AKS Sanyal R Mishra A Pandey DK. Cancer chemotherapy and beyond: Current status, drug candidates, associated risks and progress in targeted therapeutics. Genes Dis. (2023) 10:1367–401. doi: 10.1016/j.gendis.2022.02.007 PMC10310991 37397557

[6] Vaubel RA Tian S Remonde D Schroeder MA Mladek AC Kitange GJ. Genomic and phenotypic characterization of a broad panel of patient-derived xenografts reflects the diversity of glioblastoma. Clin Cancer Res. (2020) 26:1094–104. doi: 10.1158/1078-0432.CCR-19-0909 PMC7056576 31852831

[7] Catalá R Carrasco-López C Perea-Resa C Hernández-Verdeja T Salinas J. Emerging roles of LSM complexes in posttranscriptional regulation of plant response to abiotic stress. Front Plant Sci. (2019) 10:167. doi: 10.3389/fpls.2019.00167 30873189 PMC6401655

[8] Li W Deng X Chen J. RNA-binding proteins in regulating mRNA stability and translation: roles and mechanisms in cancer. Semin Cancer Biol. (2022) 86:664–77. doi: 10.1016/j.semcancer.2022.03.025 PMC9526761 35381329

[9] Mattout A Gaidatzis D Padeken J Schmid CD Aeschimann F Kalck V. LSM2–8 and XRN-2 contribute to the silencing of H3K27me3-marked genes through targeted RNA decay. Nat Cell Biol. (2020) 22:579–90. doi: 10.1038/s41556-020-0504-1 PMC7212045 32251399

[10] Zhuang H Chen B Tang C Chen X Tan W Yang L. Identification of LSM family members as novel unfavorable biomarkers in hepatocellular carcinoma. Front Oncol. (2022) 12:871771. doi: 10.3389/fonc.2022.871771 35646684 PMC9134192

[11] Sun X Zhang J Hu J Han Q Ge Z. LSM2 is associated with a poor prognosis and promotes cell proliferation, migration, and invasion in skin cutaneous melanoma. BMC Med Genomics. (2023) 16:129. doi: 10.1186/s12920-023-01564-1 37312186 PMC10262536

[12] Tomczak K Czerwińska P Wiznerowicz M. The Cancer Genome Atlas (TCGA): an immeasurable source of knowledge. Contemp Oncol (Pozn). (2015) 19:A68–77. doi: 10.5114/wo.2014.47136 PMC4322527 25691825

[13] Tang Z Li C Kang B Gao G Li C Zhang Z. GEPIA: a web server for cancer and normal gene expression profiling and interactive analyses. Nucleic Acids Res. (2017) 45:W98–W102. doi: 10.1093/nar/gkx247 PMC5570223 28407145

[14] Uhlén M Fagerberg L Hallström BM Lindskog C Oksvold P Mardinoglu A. Proteomics. Tissue-based map of the human proteome. Science. (2015) 347:1260419. doi: 10.1126/science.1260419 25613900

[15] Li T Fu J Zeng Z Cohen D Li J Chen Q. TIMER2.0 for analysis of tumor-infiltrating immune cells. Nucleic Acids Res. (2020) 48:W509–14. doi: 10.1093/nar/gkaa407 PMC7319575 32442275

[16] Gao J Aksoy BA Dogrusoz U Dresdner G Gross B Sumer SO. Integrative analysis of complex cancer genomics and clinical profiles using the cBioPortal. Sci Signal. (2013) 6:pl1. doi: 10.1126/scisignal.2004088 23550210 PMC4160307

[17] Mortazavi A Williams BA McCue K Schaeffer L Wold B. Mapping and quantifying mammalian transcriptomes by RNA-Seq. Nat Methods. (2008) 5:621–8. doi: 10.1038/nmeth.1226 PMC13303166 18516045

[18] Erlich Y Mitra PP delaBastide M McCombie WR Hannon GJ. Alta-Cyclic: a self-optimizing base caller for next-generation sequencing. Nat Methods. (2008) 5:679–82. doi: 10.1038/nmeth.1230 PMC2978646 18604217

[19] Love MI Huber W Anders S. Moderated estimation of fold change and dispersion for RNA-seq data with DESeq2. Genome Biol. (2014) 15:550. doi: 10.1186/s13059-014-0550-8 25516281 PMC4302049

[20] Putri GH Anders S Pyl PT Pimanda JE Zanini F. Analysing high-throughput sequencing data in Python with HTSeq 2.0. Bioinformatics. (2022) 38:2943–5. doi: 10.1093/bioinformatics/btac166 PMC9113351 35561197

[21] Wang ET Sandberg R Luo S Khrebtukova I Zhang L Mayr C. Alternative isoform regulation in human tissue transcriptomes. Nature. (2008) 456:470–6. doi: 10.1038/nature07509 PMC2593745 18978772

[22] Kim D Langmead B Salzberg SL. HISAT: a fast spliced aligner with low memory requirements. Nat Methods. (2015) 12:357–60. doi: 10.1038/nmeth.3317 PMC4655817 25751142

[23] Szklarczyk D Gable AL Lyon D Junge A Wyder S Huerta-Cepas J. STRING v11: protein-protein association networks with increased coverage, supporting functional discovery in genome-wide experimental datasets. Nucleic Acids Res. (2019) 47:D607–13. doi: 10.1093/nar/gky1131 PMC6323986 30476243

[24] Liu H Weng J. A comprehensive bioinformatic analysis of cyclin-dependent kinase 2 (CDK2) in glioma. Gene. (2022) 822:146325. doi: 10.1016/j.gene.2022.146325 35183683

[25] Liu H Weng J Huang CL-H Jackson AP. Is the voltage-gated sodium channel β3 subunit (SCN3B) a biomarker for glioma? Funct Integr Genomics. (2024) 24:162. doi: 10.1007/s10142-024-01443-7 39289188

[26] Chen Q Chen Y Zheng Q. The RNA-binding protein LSM family regulating reproductive development via different RNA metabolism. Biochim Biophys Acta Mol Basis Dis. (2025) 1871:167808. doi: 10.1016/j.bbadis.2025.167808 40139411

[27] Bradley RK Anczuków O. RNA splicing dysregulation and the hallmarks of cancer. Nat Rev Cancer. (2023) 23:135–55. doi: 10.1038/s41568-022-00541-7 PMC10132032 36627445

[28] Cheng Q Tang A Wang Z Fang N Zhang Z Zhang L. CALD1 modulates gliomas progression via facilitating tumor angiogenesis. Cancers (Basel). (2021) 13:2705. doi: 10.3390/cancers13112705 34070840 PMC8199308

[29] Wang J Yang Y Du B. Clinical characterization and prognostic value of TPM4 and its correlation with epithelial-mesenchymal transition in glioma. Brain Sci. (2022) 12:1120. doi: 10.3390/brainsci12091120 36138856 PMC9497136

[30] Huang K Wang H Xu J Xu R Liu Z Li Y. The tropomyosin family as novel biomarkers in relation to poor prognosis in glioma. Biology. (2022) 11:1115. doi: 10.3390/biology11081115 35892971 PMC9332389

[31] Galbo PM Madsen AT Liu Y Peng M Wei Y Ciesielski MJ. Functional contribution and clinical implication of cancer-associated fibroblasts in glioblastoma. Clin Cancer Res. (2024) 30:865–76. doi: 10.1158/1078-0432.CCR-23-0493 PMC10922678 38060213

[32] Cheon D-J Orsulic S. Mouse models of cancer. Annu Rev Pathol. (2011) 6:95–119. doi: 10.1146/annurev.pathol.3.121806.154244 20936938

[33] Ye B Fan J Xue L Zhuang Y Luo P Jiang A. iMLGAM: Integrated Machine Learning and Genetic Algorithm-driven Multiomics analysis for pan-cancer immunotherapy response prediction. iMeta. (2025) 4:e70011. doi: 10.1002/imt2.70011 40236779 PMC11995183

[34] Jahangiri L. Updates on liquid biopsies in neuroblastoma for treatment response, relapse and recurrence assessment. Cancer Genet. (2024) 288–289:32–9. doi: 10.1016/j.cancergen.2024.09.001 39241395

[35] Gonzalez T Nie Q Chaudhary LN Basel D Reddi HV. Methylation signatures as biomarkers for non-invasive early detection of breast cancer: A systematic review of the literature. Cancer Genet. (2024) 282–283:1–8. doi: 10.1016/j.cancergen.2023.12.003 38134587

